# Patient evaluation of the use of follitropin alfa in a prefilled ready-to-use injection pen in assisted reproductive technology: an observational study

**DOI:** 10.1186/1477-7827-8-111

**Published:** 2010-09-15

**Authors:** J Thomas Welcker, Frank Nawroth, Wilma Bilger

**Affiliations:** 1 Kinderwunschzentrum, Medizinisches Versorgungszentrum (MVZ) Göttingen, Göttingen, Germany; 2 Amedes Gruppe, Standort Hamburg, Fertility Center Hamburg, Hamburg, Germany; 3 Merck Serono GmbH, Darmstadt, Germany

## Abstract

**Background:**

Self-administration of recombinant human follicle-stimulating hormone (r-hFSH) can be performed using injection pen devices by women undergoing assisted reproductive technology procedures. The objective of this study was to explore the use of the prefilled follitropin alfa pen in routine assisted reproductive technology procedures in Germany.

**Methods:**

This prospective, observational study was conducted across 43 German IVF centres over a period of 1.75 years. Patients who had used the prefilled follitropin alfa pen in the current or a previous cycle of controlled ovarian stimulation completed a questionnaire to assess their opinions of the device.

**Results:**

A total of 5328 patients were included in the study. Of these, 2888 reported that they had previous experience of daily FSH injections. Significantly more patients reported that less training was required to use the prefilled follitropin alfa pen than a syringe and lyophilized powder (1997/3081 [64.8%]; p < 0.001 'less' versus 'more' training). Significantly more patients rated the prefilled follitropin alfa pen as easier in terms of use (2321/3206, 72.4%; p < 0.001 'much more easy' versus 'less easy') and daily injection (2384/3262, 73.1%; p < 0.001 'much more easy' versus 'less easy') than existing injection methods. Approximately one third of respondents rated the prefilled follitropin alfa pen as easier to use than the follitropin beta pen with reloadable cartridges. The majority (3378/4024, 83.9%) of patients had a general preference for the prefilled follitropin alfa pen over other injection methods.

**Conclusions:**

In this questionnaire-based survey, routine use of the prefilled follitropin alfa pen was well accepted and associated with favourable patient perceptions. Users of the pen found it easier to initially learn how to use, and subsequently use, than other injection methods. In general, the prefilled follitropin alfa pen was the preferred method for self-administration of gonadotrophins. Together with previous findings, the results here indicate a high level of patient satisfaction among users of the prefilled follitropin alfa pen for daily self-administration of r-hFSH.

## Background

Exogenous follicle-stimulating hormone (FSH) is used for ovulation induction and controlled ovarian stimulation (COS) during fertility treatment. Recombinant human FSH (r-hFSH) was first introduced into the market in 1995 and, unlike previously used sources of gonadotrophins, provides an unlimited, consistent, pure supply of FSH [1]. Two r-hFSH products are now commercially available - follitropin alfa and follitropin beta.

Recent developments in recombinant technology have led to improvements in the quality and consistency of gonadotrophins [1,2]. Follitropin alfa (GONAL-f^® ^filled-by-mass [FbM]) offers the most consistent and precise dosing of all FSH products [2]. This benefit may result in shorter fertility treatment regimens, a lower total FSH dose requirement, and greater consistency in the ovarian response [3-6].

As r-hFSH can be administered subcutaneously, it is suitable for self-injection by patients. r-hFSH was originally supplied in single or multidose vials or ampoules for reconstitution and injection using a syringe, but is now available in premixed solutions for use with injection-pen devices. Two injection-pen devices for the self-administration of r-hFSH are currently available. The GONAL-f^® ^(FbM) Prefilled Pen (the GONAL-f^® ^Prefilled Pen is available for use in the USA as the GONAL-f^® ^Revised Formulation Female [RFF] Pen; Merck Serono S.A. - Geneva, Switzerland, an affiliate of Merck KGaA, Darmstadt, Germany) is prefilled with premixed follitropin alfa, is ready-to-use and disposable, whereas the Puregon Pen^® ^(the Puregon Pen^® ^is available for use in Japan and the USA as the Follistim Pen^®^; Organon, Roseland, NJ, USA) is a reusable device, designed for use with premixed, prefilled loadable cartridges of follitropin beta.

An observational study was conducted in Germany over almost 2 years to explore patient perceptions of the prefilled follitropin alfa pen for the administration of r-hFSH in routine assisted reproductive technology (ART). Here, we present the results of a questionnaire-based survey to assess patients' evaluation of the pen, its perceived ease of use, and acceptability versus previously used injection methods.

## Methods

### Patients

This was a prospective, observational study conducted across 43 German IVF centres, from April 2004 until December 2005. Routine clinical data for patients undergoing cycles of COS for conventional IVF or intracytoplasmic sperm injection were entered prospectively into an electronic database (RecDate). Only one cycle per patient was included in this study.

### Assessment

When patients returned for oocyte retrieval, those who had used the prefilled follitropin alfa pen were asked to complete a multiple-choice questionnaire comparing their experience with that of other injection methods (reusable follitropin beta pen, or syringes with ampoules and vials). If the patient had used the follitropin alfa pen in a previous stimulation cycle (rather than the current cycle), she was asked to complete the questionnaire with reference to the particular cycle in which she had used the follitropin alfa pen. The questionnaire (see additional file 1) comprised eight questions specifically designed to evaluate patients' opinions on use of the r-hFSH delivery device (both initial and subsequent use) and their preferred injection method. Clinical outcomes for the ART cycles were also recorded.

### Statistical methods

Participant responses were presented as a percentage of the number of responders to the question of interest, and not the entire study population. In accordance with the design and objectives of the study, statistical evaluation was focused on the description and summary of the material obtained by means of descriptive statistics (mean, standard deviation [SD], frequency and percentage). No statistical hypotheses were specified in advance and, due to the nature of the study, the sample size was not pre-specified. In retrospective analyses, exact binomial tests were used to assess differences in the proportions of patients who gave particular responses.

## Results

### Patient and treatment characteristics

A total of 5328 patients attending the participating centres during the study period had used the prefilled follitropin alfa pen in the current or a previous cycle of COS, and were, therefore, eligible for inclusion in this study. The mean (SD) age was 33.8 (4.3) years (n = 5326); the mean (SD) body mass index was 23.4 (4.2) kg/m^2 ^(n = 5279). The mean (SD) number of days of COS per patient was 10.9 (2.7) days, with a mean (SD) cumulative dose of 2148 (993) international units of follitropin alfa per patient.

When questioned about previous experience with daily injections of FSH, 2888 respondents reported that they did have experience. The most common preparation of FSH previously used (by 52.9% of those with prior injection experience) was that of ampoules containing lyophilized powder. In total, 24.9% of patients with prior experience of daily injections with FSH had previously used the reusable follitropin beta pen.

### Initial use of the prefilled follitropin alfa pen

**Figure 1:**
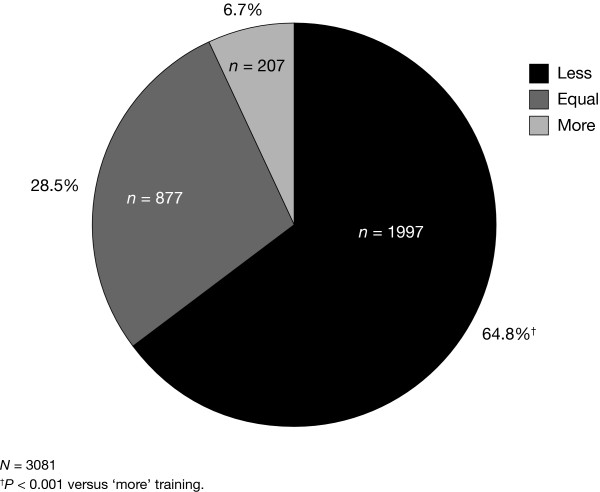
**Comparative amount of training required: prefilled follitropin alfa pen vs reconstitution and injection using a syringe**. The number and proportion of patients who reported that the prefilled follitropin alfa pen required 'less', 'equal' or 'more' training than administration (of reconstituted lyophilized powder in single- or multi-dose ampoules/vials) using a syringe. An exact binomial test was used to assess the null hypothesis that the proportion of patients who considered the pen to require less training than a syringe was equal to the proportion who considered the pen to require more training. Patients who responded that the pen and syringe required equal training were omitted from the analysis.

The majority (3967/5242, 75.7%) of respondents reported that they were trained to use the prefilled follitropin alfa pen by a nurse. The majority (52.4%) of respondents required between 5 and 9 min to be trained to use the pen (Table 1); the mean length of time required was 7 min. Significantly more respondents with relevant experience reported that less training was required to use the prefilled follitropin alfa pen compared with a syringe and lyophilized powder for injection (1997/3081 [64.8%]; p < 0.001 'less' versus 'more' training) (Fig. 1).

**Table 1:** Time required for training in use of the prefilled follitropin alfa pen

Variable	Value/number of respondents	Proportion of respondents (%)
< 5 min	734	23.4
5-9 min	1648	52.4
10-19 min	694	22.1
≥20 min	67	2.1

n = 3143.

### Routine daily use of the prefilled follitropin alfa pen

Patients who had used the prefilled follitropin alfa pen (1040 respondents) and follitropin beta pen (50 respondents) reported that they changed the pen/cartridge a mean value of 2.3 and 2.4 times per treatment cycle, respectively.

**Figure 2:**
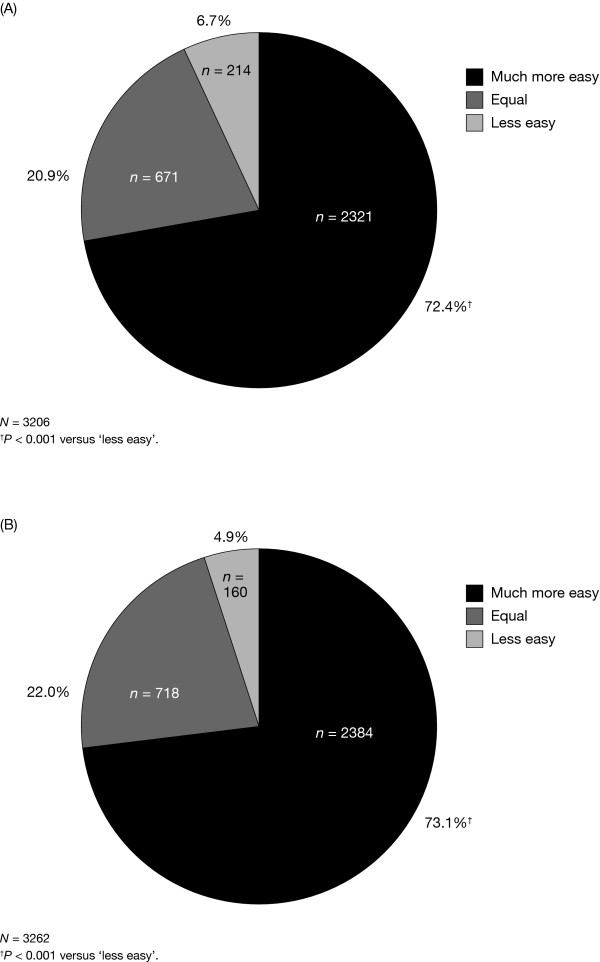
**Ease of use and daily injection: prefilled follitropin alfa pen vs existing injection methods**. The number and proportion of patients who rated (A) overall ease of use of and (B) daily injection with the prefilled follitropin alfa pen as 'much more easy', 'equal', or 'less easy' than existing injection methods. Exact binomial tests were used to test the null hypotheses that (a) the proportion of patients who rated the use of prefilled pen as much more easy than existing injections methods was equal to the proportion who rated the use as less easy and (b) the proportion of patients who rated the daily injection with the prefilled pen as much more easy than existing injections methods was equal to the proportion who rated the daily injection as less easy. Patients who responded that the pen was equally as easy to use and use daily for injection compared with existing injection methods were omitted from the analyses.

Significantly more respondents rated the overall ease of use of the prefilled follitropin alfa pen as easier than existing injection methods (2321/3206 [72.4%]; p < 0.001 'much more easy' versus 'less easy') (Fig. 2). Similarly, significantly more respondents rated daily injection with the prefilled follitropin alfa pen as easier than existing injection methods (2384/3262 [73.1%]; p < 0.001 'much more easy' versus 'less easy') (Fig. 2). Approximately 60% of respondents with experience of the reusable follitropin beta pen rated both the use of (411/660) and daily injection with (402/664) the prefilled follitropin alfa pen as easier than existing injection methods. On a scale of 1-10 (1 = less easy and 10 = much more easy) 591/1954 (30.2%) respondents gave a score of 10 when asked to evaluate use of the prefilled follitropin alfa pen compared with the reusable follitropin beta pen.

**Figure 3:**
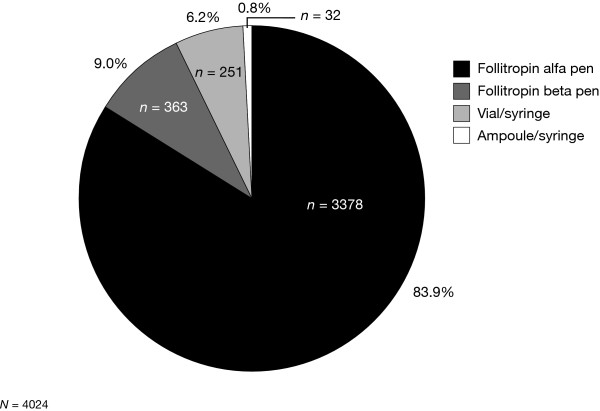
**Preferred injection methods**. The number and proportion of patients who generally preferred the prefilled follitropin alfa pen, the reusable follitropin beta pen or syringes with ampoules or vials of recombinant human follicle-stimulating hormone.

The majority (3378/4024, 83.9%) of respondents stated that they generally preferred the prefilled follitropin alfa pen when questioned about their favoured method for injection of r-hFSH (Fig. 3).

### Clinical outcomes

The mean (SD) number of oocytes retrieved per cycle was 9.75 (5.83). Over 90% of cycles resulted in embryo transfer; the clinical pregnancy rate per transfer was 32.21%. Other clinical outcomes are presented in Table 2.

**Table 2:** Clinical outcomes (N = 5328)

Outcome	
Oocyte retrieval, % (n/N)	97.58 (5199/5328)
Mean (SD) number of oocytes retrieved per cycle with oocyte retrieval*	9.75 (5.83)
Mature oocytes	
Number per cycle with oocyte retrieval, mean (SD)^†^	8.07 (4.97)
Percentage of retrieved oocytes in cycles with oocyte retrieval, mean (SD)	83.28 (19.2)
2PN oocytes	
Number per cycle with oocyte retrieval, mean (SD)^‡^	5.2 (3.69)
Percentage of inseminated/injected oocytes, mean (SD)^‡^	66.2 (27.9)
Cycles with embryo transfer, % (n/N)	90.26 (4809/5328)
Number of embryos transferred, % (n/N)	
1	10.15 (488/4809)
2	70.56 (3393/4809)
3	19.30 (928/4809)
Clinical pregnancy rate per embryo transfer, % (n/N)	32.21 (1549/4809)

*n = 5199; ^†^n = 5128; ^‡^n = 5109.

PN; pronuclear; SD, standard deviation.

Out of a total of 1549 pregnancies, there were 363 (23.43%) spontaneous miscarriages and 1076 (69.46%) live births (data were missing for 110 pregnancies). Of the 1076 births, there were 841 singleton births, 228 twin births, 6 triplet births and 1 quadruplet birth. There were 94 cases (1.95% of cycles which resulted in embryo transfer) of grade II ovarian hyperstimulation syndrome (OHSS), 22 cases (0.46%) of grade III OHSS; 28 cases of OHSS (0.58%) required hospitalization.

## Discussion

In this questionnaire-based survey, the routine use of the prefilled follitropin alfa FbM, ready-to-use injection pen was well accepted, and associated with favourable patient perceptions in 5328 ART cycles. Patients reported that use of the follitropin alfa pen required less training compared with a syringe and vials/ampoules of follitropin alfa. The prefilled pen was perceived as being easier to use than existing injection procedures/devices, including the reusable follitropin beta pen, and there was a general preference for the follitropin alfa pen.

Injection-pen devices are used to self-administer medications that require daily injection on a long-term basis. Most experience of injection-pen devices to date has been obtained in diabetic patients who self-inject insulin. Pen devices are the preferred mode of administration for insulin, as they have made the injection process easier, less painful and more convenient. This has resulted in increased patient acceptance of therapy, and has improved patient compliance with therapy and treatment outcomes, as demonstrated by improvements in glycaemic control [7-10].

Previous studies have highlighted a number of benefits for the use of pen devices for the administration of r-hFSH. Compared with the use of syringes and vials/ampoules, women undergoing fertility therapy find the use of pen devices to be less painful, less stressful, easier and more convenient, and their use is associated with greater patient satisfaction [11-14].

Results from comparative studies of the two available pen devices for injection of r-hFSH indicate a preference for the prefilled follitropin alfa pen among both patients and nurses [15,16]. In a small pilot study, patients undergoing ART procedures at a German centre were randomized to either the prefilled follitropin alfa pen or to the reusable follitropin beta pen [15]. A greater proportion of patients in the group using the prefilled follitropin alfa pen found the pen very easy to handle, and quicker to learn to use and prepare for injection than those using the reusable follitropin beta pen. Overall, the desire to continue using the device was substantially higher among patients who used the prefilled follitropin alfa pen than those who used the reusable follitropin beta pen [15].

A study of patients undergoing ART at an Australian centre compared the prefilled follitropin alfa pen with previous experience of the reusable follitropin beta pen [15,16]. Patients who had used both pen devices scored many attributes of the prefilled follitropin alfa pen more favourably than those of the reusable follitropin beta pen, including the device storage, device use and dose preparation [15]. Patients also reported less confusion with use of the prefilled follitropin alfa pen [15]. In the same study, nurses were asked to complete a questionnaire about their experiences with the prefilled follitropin alfa pen and they were also very positive about the pen [15].

A survey conducted at a centre in the USA provided questions for patients about their preferred administration device following nurse-led training in different injection-administration methods [16]. Of 94 participants who expressed a preference for a particular device, the prefilled follitropin alfa pen was the most popular (chosen by 68.1% of respondents). This was significantly more than the proportion of participants who chose the reusable follitropin beta pen as their preferred device (24.5%; p < 0.0001). In the same study, factors cited as being important for device selection included simplicity and reliability of the dosing mechanism, a design to minimize the chances of dosing errors and ease of use.

The current study is a much larger evaluation of the use of the prefilled follitropin alfa pen in routine clinical practice than those previously conducted. During the 1.75-year study period, approximately 35,000 prospective ART cycles were performed per year in Germany [17]. These data, therefore, represent 8.7% of all cycles conducted in Germany during the study period. As is typical in trials utilizing participant questionnaires, not all participants answered every question. Furthermore, participants may have been inconsistent in their answers. Although we acknowledge the limitations of this study, our results confirm the findings of previous studies, which together indicate a high level of patient satisfaction with the prefilled follitropin alfa pen for daily self-administration of r-hFSH. This may reduce the burden of fertility treatment, help to improve adherence to therapy and reduce drop-out rates [18,19].

It has been suggested that pen devices may also improve the efficacy of ovarian stimulation regimens, with better clinical outcomes associated with use of a pen device to administer r-hFSH compared with a conventional syringe [13,20,21]. However, more definitive evidence is needed, and a direct link between use of pen devices and effect on patient adherence to treatment has yet to be demonstrated. Clinical outcomes in this study were favourable, with a mean (SD) number of oocytes retrieved per cycle of 9.75 (5.83) and a clinical pregnancy rate per embryo transfer of 32.21%. As this was not designed to be a comparative study, only historical comparisons can be made between the outcomes obtained in this study with use of the prefilled follitropin alfa pen and those using other administration methods. Furthermore, such direct comparisons have inherent problems, as different study designs and study populations are used, and data are often inconsistently reported.

In summary, this study represents almost 9% of ART cycles conducted in Germany over a 1.75-year period. The results confirm previous findings on the acceptance, ease of use and preference for the prefilled follitropin alfa pen among women who self-administer r-hFSH for ART. The findings from this and previous user surveys are reflected in an audited report of pharmacy data obtained (post-study) for the 2004-2009 period which show that the majority of patients who have utilized follitropin alfa in Germany in the last 6 years have done so in the form of a prefilled pen[22].

## Competing interests

JT Welcker and F Nawroth declare that they have no competing interests. W Bilger is an employee of Merck Serono GmbH, Darmstadt, Germany, an affiliate of Merck KGaA, Darmstadt, Germany.

## Authors' contributions

All authors contributed equally to the design of the study, the collection and analysis of the data and the writing and reviewing of the manuscript. All authors have read and approved the final version of the manuscript.

## Supplementary Material

Additional file 1 **Questionnaire: comparing injection methods**. Questionnaire Click here for file
