# Supplementary material for: Patient evaluation of the use of follitropin alfa in a prefilled ready-to-use injection pen in assisted reproductive technology: an observational study
Source: Reprod Biol Endocrinol. 2010 Sep 15;8:111. doi: 10.1186/1477-7827-8-111 (PMC2949697; doi:10.1186/1477-7827-8-111)
Supplement: Additional file 1 — Questionnaire: comparing injection methods. Questionnaire [file 1477-7827-8-111-S1.DOC]

**Questionnaire: comparing injection methods**

1. Did you receive in the previous or current stimulation cycle gonadotrophin formulation in a ready-to-use prefilled pen?

- Yes
- No

1. Did you have experience with daily injections prior to this treatment cycle?

- Yes
  - Ampoules with lyophilized powder
  - Vials with lyophilized powder
  - Reusable pen with cartridge to be loaded
  - Vials with liquid ready to use
- No

1. Who explained to you the handling of the injection with the prefilled ready-to-use pen?

- Physician
- Nurse
- Patient herself

1. (a) How much patient training does this pen require in comparison to previous lyophilized single or multidose injections?

- More training
- Equal training
- Less training
- Patient had no experience

1. How much time did the training require?

Enter minutes ___

1. How often did you have to change the pen or the cartridge during the treatment cycle?

- Once
- Twice
- Three times
- Four times
- Five times

1. (a) How did you evaluate the usage of the ready-to-use pen in comparison to previous formulations?

- Much more easy
- Equal
- Less easy
- NA

(b) How did you evaluate the daily injection of the pen in comparison to previous formulations?

- Much more easy
- Equal
- Less easy
- NA

1. How did you evaluate the ready-to-use prefilled pen compared with the pen with cartridges to be loaded?

- Scale of 1 to 10 (1 = less easy, 5–6 = equal, 10 = much more easy)
- NA

1. Which kind of packaging or formulation do you prefer in general?

- Glass ampoule
- Vial/syringe
- Ready-to-use pen
- Reusable pen with cartridges to be loaded
- NA
